# Obesity-Related Hemodynamic Alterations in Patients with Cushing’s Disease

**DOI:** 10.3390/jcm13061658

**Published:** 2024-03-14

**Authors:** Agnieszka Jurek, Paweł Krzesiński, Beata Uziębło-Życzkowska, Przemysław Witek, Grzegorz Zieliński, Robert Wierzbowski, Anna Kazimierczak, Małgorzata Banak, Grzegorz Gielerak

**Affiliations:** 1 Department of Cardiology and Internal Medicine, Military Institute of Medicine—National Research Institute, 04-141 Warsaw, Poland; pkrzesinski@wim.mil.pl (P.K.); buzieblo-zyczkowska@wim.mil.pl (B.U.-Ż.);; 2 Department of Internal Medicine, Endocrinology, and Diabetology, Medical University of Warsaw, 02-091 Warsaw, Poland; 3 Department of Neurosurgery, Military Institute of Medicine—National Research Institute, 04-141 Warsaw, Poland; gzielinski@wim.mil.pl

**Keywords:** Cushing’s disease, global longitudinal strain, impedance cardiography, metabolic syndrome, obesity, hemodynamic profile, cardiovascular complications

## Abstract

**Background**: Cushing’s disease (CD) is associated with a specific form of metabolic syndrome that includes visceral obesity, which may affect cardiovascular hemodynamics by stimulating hypercortisolism-related metabolic activity. The purpose of this study was to evaluate the relationship between obesity and the hemodynamic profile of patients with CD. **Methods**: This prospective clinical study involved a hemodynamic status assessment of 54 patients newly diagnosed with CD with no significant comorbidities (mean age of 41 years). The assessments included impedance cardiography (ICG) to assess such parameters as stroke index (SI), cardiac index (CI), velocity index (VI), acceleration index (ACI), Heather index (HI), systemic vascular resistance index (SVRI), and total arterial compliance index (TACI) as well as applanation tonometry to assess such parameters as central pulse pressure (CPP) and augmentation index (AI). These assessments were complemented by echocardiography to assess cardiac structure and function. **Results**: Compared with CD patients without obesity, individuals with CD and obesity (defined as a body mass index ≥ 30 kg/m^2^) exhibited significantly lower values of ICG parameters characterizing the pumping function of the heart (VI: 37.0 ± 9.5 vs. 47.2 ± 14.3 × 1*1000^−1^*s^−1^, *p* = 0.006; ACI: 58.7 ± 23.5 vs. 76.0 ± 23.5 × 1/100/s^2^, *p* = 0.005; HI: 11.1 ± 3.5 vs. 14.6 ± 5.5 × Ohm/s^2^, *p* = 0.01), whereas echocardiography in obese patients showed larger heart chamber sizes and a higher left ventricular mass index. No significant intergroup differences in blood pressure, heart rate, LVEF, GLS, TACI, CPP, or AI were noted. **Conclusions**: Hemodynamic changes associated with obesity already occur at an early stage of CD and manifest via significantly lower values of the ICG parameters illustrating the heart’s function as a pump, despite the normal function of the left ventricle in echocardiography.

## 1. Introduction

Cushing’s disease (CD), caused by a pituitary neuroendocrine tumor, leads to a specific type of metabolic syndrome that includes hypertension, obesity, impaired glucose metabolism, and dyslipidemia [1,2,3]. Chronic hypercortisolemia in patients with CD results in the excessive accumulation of visceral fat due to abnormal adipokine production [4]. Visceral obesity plays an important role in hypercortisolism-induced metabolic abnormalities and increased activity of the renin–angiotensin–aldosterone system activity in patients with CD [1,2,3,4,5]. Visceral obesity in patients with CD not only contributes to metabolic syndrome, but it is also an independent risk factor for cardiovascular disease [1,3,6,7]. Importantly, the structure and function of adipose tissue in patients with CD differ from those of healthy individuals [1,8,9]. The various hypercortisolism-induced metabolic abnormalities occurring in obese patients with CD may affect cardiovascular hemodynamics. There are no data on the effect of obesity on the hemodynamic profile of patients with CD and also few data are known on the association between obesity and hemodynamic disturbances in people without CD [10,11]. It was shown that the hemodynamic profile of a person with obesity is characterized by increased cardiac output and thoracic fluid content and decreased vascular resistance in comparison with these parameters in healthy individuals [12].

More studies are needed to enhance our understanding of the pathophysiology of CD-related obesity as a modifiable cardiovascular risk factor, in order to develop effective preventive and therapeutic strategies. Unfortunately, subclinical consequences of hypercortisolism in newly diagnosed patients with early CD, particularly with comorbid obesity, may be undetectable with standard methods. Therefore, novel and easy-to-use diagnostic methods would be of additive value to the standard methods of assessing cardiovascular structure and function in patients with CD. A detailed evaluation of the nature of obesity in patients with CD by innovative noninvasive diagnostic methods, such as impedance cardiography (ICG), applanation tonometry (AT), and echocardiographic assessment of global longitudinal strain (GLS), may provide additional data on cardiovascular hemodynamics, particularly the heart’s pumping function, preload, and afterload [13,14,15,16,17,18]. Our previous studies demonstrated the usefulness of ICG in identifying subclinical cardiovascular complications in patients with CD [19,20].

The purpose of this analysis was to assess the relationship between obesity and the hemodynamic profile of patients newly diagnosed with CD with no significant comorbidities.

## 2. Materials and Methods

### 2.1. Study Population

This was a prospective observational cohort study involving a comprehensive assessment of 54 patients (mean age of 41 years) newly diagnosed with CD with no significant comorbidities (although 64.8% were diagnosed with hypertension). These patients were admitted to the Military Institute of Medicine—National Research Institute between 2016 and 2021 in order to undergo a thorough cardiovascular assessment prior to transsphenoidal pituitary neuroendocrine tumor resection surgery.

This study was approved by the ethics committee at the Military Institute of Medicine—National Research Institute (approval No. 76/WIM/2016) and compliant with the Declaration of Helsinki and Good Clinical Practice guidelines. Each patient received detailed information on the purpose of this study and signed an informed consent form. This study was financed by the Polish Ministry of Research and Higher Education/Military Institute of Medicine—National Research Institute in Warsaw (grant No. 453/WIM).

### 2.2. Inclusion Criteria

The diagnosis of CD was established based on the presence of the typical (clinical and hormonal) evidence of hypercortisolism with no adrenocorticotropic hormone (ACTH) response to corticotropin-releasing hormone (CRH) stimulation, which meets the current guidelines for the diagnosis and treatment of CD [21,22,23]. Physical examination findings consistent with the signs and symptoms of CD, including central obesity with the characteristic altered body fat distribution (a moon face and a short, thick neck); muscle atrophy in the torso and limbs; purplish stretch marks on the abdomen, hips, and thighs; thinned skin; ecchymoses; signs and symptoms of hyperandrogenism; bone pain; frequent infections; erectile dysfunction in men; and secondary amenorrhea and infertility in women. Hormone test results included elevated 24 h urinary free cortisol levels, increased morning serum cortisol levels, altered circadian rhythmicity of ACTH and cortisol secretion, elevated or detectable morning serum ACTH, and a lack of overnight serum cortisol suppression to <1.8 mg/dL during a low-dose dexamethasone suppression test (1 mg or 2 mg of dexamethasone administered at midnight). In order to ensure a pituitary etiology of CD, all patients underwent a two-day high-dose (2 mg every 6 h = a total of 8 mg) dexamethasone suppression test (HDDST), which was expected to show low serum cortisol or a >50% decrease in urinary-free cortisol levels. Moreover, each patient was shown to have no ACTH secretion response to a CRH stimulation test (with 100 μg intravenous CRH), and the presence of a pituitary neuroendocrine tumor was confirmed via contrast magnetic resonance imaging of the pituitary. Patients with inconclusive hormone tests or imaging studies additionally underwent bilateral inferior petrosal sinus sampling (used to determine ACTH levels in the venous blood before and after CRH stimulation) [21,22,23].

### 2.3. Exclusion Criteria

The following comorbidities, which might considerably affect hemodynamic profiles, constituted our study exclusion criteria: (1) heart failure with mildly reduced or reduced left ventricular ejection fraction (LVEF) (i.e., LVEF of <50%); (2) cardiomyopathy; (3) clinically significant valvular heart disease or arrhythmia; (4) coronary artery disease, including a history of acute coronary syndrome; (5) a poor acoustic window on echocardiography; (6) a history of pulmonary embolism; (7) a history of a stroke or transient ischemic attack; (8) renal failure (estimated glomerular filtration rate < 60 mL/min/1.73 m^2^); (9) peripheral vascular disease and polyneuropathy; (10) chronic obstructive pulmonary disease; (11) respiratory failure (decreased partial pressure of arterial oxygen [PaO_2_] < 60 mmHg and/or increased partial pressure of carbon dioxide [PaCO_2_] > 45 mmHg); (12) a history of head trauma; (13) pregnancy; (14) age < 18 years; (15) no written informed consent.

### 2.4. Additional Hormone Tests

Due to the fact that hypercortisolemia inhibits gonadotropin release, hormone testing was expanded to include follicle-stimulating hormone and luteinizing hormone levels. The patients also had their serum thyroid-stimulating hormone levels tested to determine possible hypothyroidism, associated with reduced CRH and thyroid-stimulating hormone secretion and hypercortisolism-induced alterations in thyroid function. The patients with CD included in this study were not receiving any medications affecting the hypothalamus–pituitary–adrenal axis. None of the female patients with CD were pregnant at the time of the study or had given birth within the previous five years.

### 2.5. Laboratory Tests

In order to detect possible metabolic conditions, such as impaired fasting glucose, type 2 diabetes mellitus, or dyslipidemia, all patients underwent fasting blood tests from venous blood samples collected in the morning (at 6:00 a.m.). The tests evaluated the levels of fasting glucose, creatinine, eGFR, total cholesterol, low-density lipoprotein cholesterol, high-density lipoprotein cholesterol, and triglycerides, as well as a complete blood count.

### 2.6. Anamnesis and Physical Examination

The patients were thoroughly evaluated for cardiovascular risk factors, cardiovascular signs and symptoms, a family history of cardiovascular disease, comorbidities, prescription medications and other drugs, and smoking.

The body mass index (BMI) was calculated, and obesity was determined based on the International Diabetes Federation and European Society of Cardiology guidelines, which define it as a BMI of ≥30 kg/m^2^ [24,25]. In the study, patients were divided into two groups: patients with CD and obesity (defined as high body mass index ≥ 30 kg/m^2^) and patients with CD without obesity (defined as normal BMI < 30 kg/m^2^).

Physical examination included the resting heart rate (HR), systolic and diastolic blood pressure, and anthropometric parameters.

Office blood pressure measurements were taken by a trained nurse in seated patients in the morning, after a 5 min rest. The blood pressure monitor used was Omron M4 Plus (Omron Healthcare Co. Ltd., Kyoto, Japan), which meets the European Society of Cardiology criteria [26].

### 2.7. Echocardiography

Two-dimensional echocardiography included standard parasternal, apical, and subcostal views with a 2.5 MHz transducer (VIVID E95, GE Medical System, Wauwatosa, WI, USA) in accordance with the American Society of Echocardiography (ASE) and the European Association of Cardiovascular Imaging (EACVI) guidelines [27]. The parasternal long-axis view was used to measure the left ventricular end-diastolic diameter (LVEDd), right ventricular end-diastolic diameter (RVEDd), interventricular septal thickness, and left atrial (LA) diameter. Linear 2-dimensional left ventricular measurements were used to calculate the left ventricular mass index (LVMI), which is the left ventricular mass divided by the body surface area (LVMI cut-off values of >115 g/m^2^ for men and >95 g/m^2^ for women meet ASE and EACVI criteria for the diagnosis of left ventricular hypertrophy). The LVEF was calculated with the biplane Simpson method, based on 2-dimensional views of the left ventricle during systole and diastole in four- and two-chamber apical views. The ascending aortic diameter, valvular structure and function, and pericardium were assessed. The patients were assessed for left ventricular diastolic dysfunction according to current guidelines. Pulse wave Doppler in an apical four-chamber view aligned with mitral valve tips was used to visualize mitral inflow, including the early passive blood inflow (E) and the later atrial (A) contribution to the mitral inflow, E/A ratio, and early mitral inflow deceleration time. Apical four-chamber views were used to determine the septal and lateral early diastolic mitral annular velocities (e′ avg), and the E/e′ avg ratio was calculated [27,28].

Global longitudinal strain (GLS) was assessed via electrocardiography-gated automated function imaging in two-, three-, and four-chamber views. The rates of >60 frames per second were used for optimal speckle-tracking strain assessment. Patients with a poor acoustic window were excluded from the study. Semiautomated endocardial border detection was initiated by manually selecting two points identifying the mitral annulus and one point at the apex. Segmental and whole-chamber strain was assessed. The results have been presented in the form of a “bull’s eye” graph. The data were analyzed for four-, three-, and two-chamber views, and average GLS was calculated [29].

### 2.8. Impedance Cardiography

Based on the phenomenon of impedance variability in individual body segments associated with regional arterial blood flow, ICG is a noninvasive tool for assessing cardiovascular hemodynamics. ICG assessments were conducted by a trained nurse with a Niccomo device (Medis, Ilmenau, Germany) in patients who had been resting for 10 min in a supine position. ICG data were recorded during a 10 min assessment and processed with dedicated software (Niccomo Software, Medis). We analyzed the mean values of the following hemodynamic parameters reflecting the pumping function of the heart: (1) stroke volume (SV [mL]) and stroke index (SI [mL/m^2^]), based on the following formula: SV = VEPT × (dZmax/Z0) × LVET, where VEPT is tissue volume calculated from body weight, height, and patient sex, Z0 is the initial thoracic impedance, dZmax is the maximum change in thoracic impedance, and LVET is the left ventricular ejection time; (2) cardiac output (CO [mL] = SV × HR), and cardiac index (CI [mL*m^−2^*min^−1^]); (3) velocity index (VI [1*1000^−1^*s^−1^]); (4) acceleration index (ACI [1/100/s^2^], which is the peak acceleration of blood flow in the aorta; and (5) Heather index (HI [Ohm/s^2^] = dZmax × TRC, where TRC the time interval between the R-peak in the electrocardiogram and the C-point on the impedance wave). We also conducted a detailed analysis of the following afterload parameters: (1) systemic vascular resistance (SVR [dyn*s*cm^−5^]) together with SVR index (SVRI [dyn*s*cm^−5^*m^2^]) and (2) total arterial compliance (TAC) and TAC index (TACI [mL/mmHg] = SV/pulse pressure [mL/mmHg*m^2^]). Preload was assessed based on thoracic fluid content (TFC [1/kOhm], based on the formula TFC = 1000/Z0, where Z0 is the initial thoracic impedance [30,31,32].

### 2.9. Applanation Tonometry

Applanation tonometry is a novel method of indirectly illustrating arterial pressure waveform in the aorta and arterial stiffness, which reflect left ventricular afterload. AT parameters were assessed noninvasively with a SphygmoCor system (AtCor Medical, Sydney, NSW, Australia). The measurements were taken in supine patients by a qualified nurse immediately after ICG. Radial artery pressure curves were recorded via AT with a micromanometer (Millar Instruments, Houston, TX, USA) strapped onto the left wrist. We selected high-quality recordings for our analysis. Radial pulse was calibrated against the latest brachial systolic and diastolic blood pressure measurement with an oscillometric module of the Niccomo device. SphygmoCor software (version 9.0; AtCor Medical Inc. Pty Ltd., Sydney, NSW, Australia) was used to process the arterial waveform and generate an appropriate aortic blood pressure curve from the radial pulse curve. The analyzed waveforms were composed of the pulse wave generated by the aorta and were augmented by an overlapping reflected wave. Our analyses yielded the following parameters: central systolic blood pressure; central diastolic blood pressure; central pulse pressure (CPP); augmentation pressure, which is the absolute increase in aortic systolic pressure (directly generated by left ventricular contraction) resulting from the reflection wave; and the augmentation index, calculated as AP × 100/CPP, which is a quotient of the augmentation pressure and the blood pressure in the aorta [33].

### 2.10. Statistical Analysis

For the statistical analysis of the results, we used MS Office Excel 2023 and Statistica 12.0 (StatSof Inc., Tulsa, OK, USA). Data distribution and normality were assessed visually on histograms and with the use of the Kolmogorov–Smirnov test. Continuous variables were expressed as mean ± standard deviation (SD) or median (interquartile range, IQR), and categorical variables were expressed as absolute and relative (percentage) values. In order to evaluate differences between the subgroups of CD patients with and without comorbid obesity, we used Student’s *t*-test for normally distributed data, and the Mann–Whitney U test for non-normally distributed data. A comparative analysis with the use of the Mann–Whitney U test was conducted on the data from patients stratified into two subgroups: patients with CD and obesity (BMI ≥ 30 kg/m^2^, *n* = 22) and patients with CD without obesity (BMI < 30 kg/m^2^, *n* = 32). The relationship between selected indices of cardiovascular function and obesity (represented as BMI) was analyzed separately for each one in a multivariable regression model, adjusting for age and hypertension as potential covariates related to hemodynamics. The threshold of statistical significance was adopted at *p* < 0.05.

## 3. Results

### 3.1. Baseline Characteristics

Nearly half of the patients with CD were found to be obese (*n* = 22, 40.7%). Overall, 20 of the 54 patients with Cushing’s disease (37%) were diagnosed with type 2 diabetes mellitus, 5 (9.3%) had prediabetes, and 29 (46.3%) had normal glucose tolerance. Of the patients with Cushing’s disease and type 2 diabetes, 14 received metformin, 5 received metformin with insulin, and 1 received insulin.

The mean age, HR, hemoglobin, creatinine, and sex distribution were similar in the subgroup with and without obesity (Table 1).

### 3.2. Echocardiographic Assessment

Patients with CD and obesity (BMI ≥ 30 kg/m^2^) showed larger dimensions of heart chambers and ascending aorta (RVEDd, *p* < 0.001; LVEDd, *p* = 0.028; LA diameter, *p* < 0.001; aortic arch, *p* = 0.005) and higher rates of left ventricular mass index (LVMI, *p* = 0.028). We observed no significant differences between the subgroups in terms of the systolic (LVEF or GLS) or diastolic function of the left ventricle (Table 1).

### 3.3. ICG and AT Assessment

The most noticeable differences in ICG were observed for parameters of the left ventricular function as a pump. In obese individuals, VI (*p* = 0.006), ACI (*p* = 0.005), and HI (*p* = 0.012) were lower, whereas the systolic time ratio (STR) was higher (*p* = 0.038) than those in non-obese individuals, with SI and CI comparable in both subgroups. We observed no significant differences in afterload (TACI, SVRI, CPP, or augmentation index) or preload (TFC) parameters (Table 1).

### 3.4. Correlation Analysis

Analyzing the relationships between BMI and ICG hemodynamic parameters, we observed significant correlations, independent of sex and hypertension, between BMI and CI (R = 0.46; *p* < 0.001), SI (R = 0.29; *p* = 0.043), SVRI (−0.31; 0.028), and VI (R = −0.37; *p* = 0.0006)—see Table 2.

## 4. Discussion

The results of our study revealed a relationship between obesity and hemodynamic profile assessed via ICG in patients newly diagnosed with active CD. The use of novel diagnostic modalities demonstrated that excessive fat accumulation in young and middle-aged patients with CD, already at the early stages of the disease, is associated with some hemodynamic changes in the cardiovascular system, which—at that stage—may still be undetectable in routine assessments. These findings support the need for the early detection of subclinical heart dysfunction in patients with CD to enable early treatment and help prevent cardiovascular complications [1,34,35,36].

Occurring in 25%–100% of patients with CD, visceral obesity is one of the most common components of metabolic syndrome, often being the first sign of the disease. The duration of hypercortisolism correlates with obesity development [1,7,37,38], with chronic excessive cortisol levels being responsible for the abnormal distribution of adipose tissue [39]. The mechanisms behind this phenomenon may be due to the tissue overexpression of the 11β-hydroxysteroid dehydrogenase type 1 (11β-HSD1), which affects the pattern of excessive fat distribution in the torso, face, and neck [1,6]. Visceral obesity found in patients with CD is not only a component of metabolic syndrome but is in itself associated with increased metabolic activity, which makes it an independent cardiovascular risk factor, leading to the development of cardiovascular disease [1,4,9]. The tendency to accumulate visceral fat in patients with CD is also associated with abnormal adipokine production [4,6,40,41].

Our study included patients newly diagnosed with active CD with no clinically significant cardiovascular disease. Males were underrepresented in both subgroups. The proportion of patients with hypertension was 64.8%, which is comparable with that reported by other authors [38,42,43,44] and similarly distributed between subgroups. However, the patients in our study presented well-controlled hypertension (mean blood pressure was 126/83 mmHg), usually with one or two medications. Considering both sex and hypertension as potential confounders, these variables were included in regression models evaluating correlations between hemodynamics and BMI.

Similar to reports by other authors, our study showed higher SV and CO values in obese patients with CD; however, the respective indexed values (SI and CI) were comparable in obese and non-obese patients [12,45]. A more detailed ICG assessment demonstrated significant impairment of the pumping function of the heart as evidenced by lower HI, VI, and ACI values, and a higher STR value. The analysis of correlations revealed the independence of age and sex interrelation between some hemodynamic indices (CI, SI, SVRI, VI) and BMI. The paradox of the positive relation of obesity with volume indices of left ventricular function (CI and SI), which is negative with the marker of both its outflow and myocardial contractility (VI) encourages further studies investigating the (patho)physiological background of this phenomenon.

These findings were detected despite the lack of echocardiographic evidence of left ventricular systolic or diastolic dysfunction.

Moreover, our study showed larger heart chamber diameters and significantly higher LVMI in patients with CD and obesity, which is consistent with numerous earlier reports by other authors [46,47,48]. Nonetheless, it seems that in this case, increased heart chamber size and left ventricular hypertrophy should not be considered as only secondary to an increase in body weight. Hypercortisolism in patients with CD worsens the structural and functional condition of the heart muscle and may lead to myocardial fibrosis [48]. This results in myocardial remodeling associated with concentric left ventricular hypertrophy, which may impair left ventricular hemodynamic function, subsequently leading to myocardial dysfunction and symptomatic heart failure [49,50,51]. The effective treatment of patients with CD has been shown to normalize their serum cortisol levels and ultimately stop myocardial remodeling [47]. Therefore, the ICG-evidenced impaired pumping function of the heart may result from myocardial remodeling associated with complex metabolic and neuroendocrine changes in obese patients with CD [52]. These findings are consistent with previous reports on the adverse effect of obesity on left ventricular contractility [53,54,55,56].

The potential mechanisms underlying the results of our study remain to be elucidated. An interesting perspective is represented by the cross-talk between glucocorticoid (GR) and mineralocorticoid receptors (MR) and their impact on metabolic syndrome. Excessive activation of the MR in extra-renal tissues by aldosterone or glucocorticoids depending on the expression of 11beta-hydroxysteroid dehydrogenase type 2 has been shown to be associated with the development of vascular dysfunction and metabolic abnormalities, leading to obesity and metabolic syndrome. High concentrations of aldosterone may also activate the transcriptional function of the GR. These mechanisms result in an interaction between GR and MR in the regulation of adipogenesis [57].

The novelty of our approach is due to the use of noninvasive tools (ICG, AT) for hemodynamic assessment of the cardiovascular system in patients with CD to detect subclinical changes associated with obesity. On the one hand, our findings support earlier observations in other patient groups; on the other hand, they cast a new light on the relationship between obesity and an impaired hemodynamic profile in CD, which may result in the early development of cardiovascular complications.

### 4.1. Clinical Implications

We determined that a dysfunctional pumping action of the heart is the key marker of impaired cardiovascular hemodynamics in obese patients newly diagnosed with CD. The use of noninvasive diagnostic methods in this study revealed a complex relationship between obesity-related hemodynamic changes and the efficiency of left ventricular contractions. An early assessment of a patient’s hemodynamic profile may help detect subclinical cardiovascular dysfunction. Such a personalized approach may facilitate early therapeutic intervention and monitoring of treatment effectiveness focused on preventing myocardial remodeling and heart dysfunction.

### 4.2. Limitations

One limitation of our study was the small sample size. This was a result of the relatively low incidence of pituitary neuroendocrine tumors secreting ACTH. The exclusion of patients with clinically significant comorbidities further diminished the study population. However, this helped to eliminate the effect of additional factors on hemodynamic profiles. The patients assessed in our study were mostly young and middle-aged individuals with CD; therefore, our conclusions should not be extrapolated to older subjects. Although we conducted neither cardiac stress tests nor coronary angiography to exclude asymptomatic ischemic heart disease, other thorough assessments showed no physical, electrocardiographic, or echocardiographic evidence suggesting myocardial ischemia. Another potential limitation of our study is the fact that some patients had hypertension; however, it was well controlled with medications. The hemodynamic assessments involved the use of noninvasive methods as an alternative to the more expensive and less readily available invasive techniques. Nonetheless, we acknowledge the fact that noninvasive measurements can only provide indirect measurements and depend on the patient’s condition, which may vary over time.

## 5. Conclusions

The results of our study support the usefulness of ICG in diagnosing early heart dysfunction associated with obesity in patients with CD. Asymptomatic impairment of the heart’s pumping function seems to be the earliest clinical sign of cardiovascular hemodynamic abnormalities, which at this stage are still undetectable with standard echocardiography. Individual hemodynamic profile assessment with novel noninvasive diagnostic methods encourages further studies on cardiovascular system function in obese individuals with CD and on the use of personalized therapies, which aim at preventing adverse cardiovascular events.

## Figures and Tables

**Table 1:** Clinical, echocardiographic, hemodynamic, and applanation tonometry variables in patients with Cushing’s disease (CD) and with or without obesity.

Variables	Patients with CD and Obesity (BMI ≥ 30 kg/m^2^) Mean ± SD/(Median (IQR) or *n* (%)	Patients with CD without Obesity (BMI < 30 kg/m^2^) Mean ± SD Median (IQR) or *n* (%)	*p*-Value
**Clinical variables**			
**Age**	49.0 (31.0–55.0)	38.9 (33.0–44.5)	0.283
**Males**	7 (31.8)	5 (15.6)	0.160
**BMI**	36.30 ± 4.30	25.18 ± 2.79	<0.0001
**Hypertension**	15 (68.2)	20 (62.5)	0.667
**Hemoglobin**	14.66 ± 1.15	14.43 ± 1.36	0.698
**Creatinine**	0.80 (0.70–0.90)	0.86 (0.80–0.90)	0.876
**Echocardiographic variables**			
**RVEDd** [mm]	30.87 ± 3.21	27.33 ± 3.75	<0.0001
**LVEDd** [mm]	48.87 ± 3.50	46.24 ± 4.50	0.028
**LA diameter** [mm]	38.92 ± 3.71	34.60 ± 3.17	<0.0001
**AAoD** [mm])	32.08 ± 3.37	29.21 ± 3.51	0.0049
**LVMI** [g/m^2^)]	103.48 ± 23.95	89.68 ± 19.85	0.028
**LVH**	10 (32.3)	10 (47.6)	0.264
**LVEF** [%] (Simpson method)	65.55 ± 4.78	65.88 ± 3.73	0.786
**GLS** [%]	18.00 ± 2.36	18.09 ± 2.35	0.898
**e’avg**	7.75 (7.00–12.00)	9.74 (8.00–12.00)	0.385
**E/e’avg**	8.14 ± 2.61	7.33 ± 1.52	0.169
**E/A**	0.98 ± 0.27	1.16 ± 0.35	0.058
**Impedance cardiography variables**			
**HR** [bpm]	73.86 ± 12.40	69.67 ± 11.50	0.214
**SBP** [mmHg]	125.5 (118.0–138.0)	122.47 (111.0–132.0)	0.113
**DBP** [mmHg]	81.5 (77.0–85.0)	82.83 (74.0–92.0)	0.993
**MBP** [mmHg]	95.0 (87.0–97.0)	92.91 (83.5–101.5)	0.573
**PP** [mmHg]	45.0 (37.0–57.0)	39.7 (34.0–44.0)	0.007
**SI** [mL/m^2^]	42.48 ± 12.47	41.80 ± 8.84	0.821
**SV** [mL]	87.9 ± 29.0	73.9 ± 17.1	0.034
**CI** [mL*m^−2^*min^−1^]	3.15 ± 0.86	2.89 ± 0.71	0.234
**CO** [mL*min^−1^]	6.39 ± 1.86	5.09 ± 1.36	0.006
**SVRI** [dyn*s*cm^−5^*m^2^]	2456.82 ± 792.96	2557.40 ± 705.18	0.632
**TACI** [mL/mmHg*m^2^]	0.95 ± 0.34	1.09 ± 0.30	0.118
**VI** [1*1000^−1^*s^−1^]	39.5 (33.0–43.0)	47.17 (35.0–56.0)	0.006
**ACI** [1/100/s^2^]	65.0 (42.0–70.0)	76.0 (55.0–90.0)	0.005
**HI** [Ohm/s^2^]	11.10 ± 3.48	14.58 ± 5.52	0.012
**STR** [%]	0.41 ± 0.10	0.35 ± 0.09	0.038
**TFC** [1/kOhm]	26.05 ± 3.82	25.17 ± 4.00	0.428
**Applanation tonometry**			
**CSBP** [mmHg]	116.00 ± 12.08	116.00 ± 18.49	1.000
**CDBP** [mmHg]	82.0 (77.0–85.0)	84.92 (76.0–92.0)	0.572
**CPP** [mmHg]	31.0 (27.0–40.0)	31.08 (25.0–35.0)	0.478
**Augmentation pressure**	7.50 (2.50–11.50)	8.31 (5.00–11.0)	0.706
**Aortic Alx (AP/PP) = AI** [%]	21.15 ± 14.27	24.58 ± 9.44	0.333

**AAoD**—ascending aortic diameter; **ACI**—acceleration index; **AI**—augmentation index; **AP**—augmentation pressure; **BMI**—body mass index; **CI**—cardiac index; **CD**—Cushing’s disease; **CDBP**—central diastolic blood pressure; **CO**—cardiac output; **CSBP**—central systolic blood pressure; **CPP**—central pulse pressure; **DBP**—diastolic blood pressure; **E/A**—early-to-late diastolic mitral inflow velocity ratio; **e’avg**—early diastolic mitral annular velocity; **E/e’avg**—early mitral inflow to early mitral annular velocity ratio; **GLS**—global longitudinal strain; **HI**—Heather index; **HR**—heart rate; **IQR**—interquartile range; **LA**—left atrial; **LVEF**—left ventricular ejection fraction; **LVEDd**—left ventricular end-diastolic diameter; **LVH**—left ventricular hypertrophy; **LVMI**—left ventricular mass index; **MBP**—mean blood pressure; **PP**—pulse pressure; **RVEDd**—right ventricular end-diastolic diameter; **SBP**—systolic blood pressure; **SI**—stroke index; **STR**—systolic time ratio; **SVRI**—systemic vascular resistance index; **TACI**—total arterial compliance index; **TFC**—thoracic fluid content; **VI**—velocity index.

**Table 2:** Correlations between hemodynamic parameters assessed with impedance cardiography and body mass index, adjusted for sex and hypertension in multivariable regression models.

	BMI	*p*-Value
**CI** [mL*m^−2^*min^−1^]	0.46	<0.001
**SI** [mL/m^2^]	0.29	0.043
**SVRI** [dyn*s*cm^−5^*m^2^]	−0.31	0.028
**TACI** [mL/mmHg*m^2^]	−0.05	0.679
**VI** [1*1000^−1^*s^−1^]	−0.31	0.026
**ACI** [1/100/s^2^]	−0.21	0.112
**HI** [Ohm/s^2^]	−0.19	0.155
**STR** [%]	0.09	0.515
**TFC** [1/kOhm]	0.03	0.818

**ACI**—acceleration index; **BMI**—body mass index; **CI**—cardiac index; **HI**—Heather index; **SI**—stroke index; **STR**—systolic time ratio; **SVRI**—systemic vascular resistance index; **TACI**—total arterial compliance index; **TFC**—thoracic fluid content; **VI**—velocity index.

## Data Availability

The data presented in this study are available upon request from the corresponding author. The data are not publicly available due to privacy or ethical restrictions.
